# Prayer, fasting, and mental health: a very important question

**DOI:** 10.1192/j.eurpsy.2026.11500

**Published:** 2026-07-31

**Authors:** Z. Souhlal

**Affiliations:** New Hospital, Vernon, France

## Abstract

**Introduction:**

Regular prayer in religious traditions such as Islam and Christianity is not only a spiritual practice but also has measurable neurobiological effects on the brain and body.

Neuroimaging and physiological studies show that prayer—especially with focused attention and breath control—activates brain regions associated with calm, emotional regulation, and relaxation, including the prefrontal cortex, limbic system, and autonomic nervous system.

**Objectives:**

The relationship between prayer, fasting, and psychiatry is multidimensional, involving both psychological and neurobiological mechanisms that can have significant impacts on mental health. Religious practices such as regular prayer and ritual fasting (e.g., Ramadan in Islam or fasting periods in Christianity) are increasingly recognized for their benefits in emotional regulation, stress management, and even cognitive performance

**Methods:**

Neurobiology of prayer and fasting on mental health The neurobiology of prayer and fasting on mental health is a fascinatingarea of study that explores the physiological and psychological effects of these spiritual practices.

The review of litterature and different researchs

**Results:**

Prayer, particulary regular prayer practiced in religious traditions like islam, Christianity, a,d others, is often perceived as a means of spiritual connection, but it also hasmeasurable effects on the brain.

Activation of brain regions associated with relaxation :Prayer canactivate brain regions related to relaxation and calm, such as the prefrontal cortex and the limbic system (wich regulates emotions) These area are involved in stress management, emotional regulationand activation of feelings of inner peace Reduction in activation on the stress system:Meditativepractices like prayer can modulate the hypothalamic -pituitary-adrenal (HPA) axis, wich is responsible for the stress response.

**Conclusions:**

Reducing stress, decreasing inflammation, and promoting neurogenesis, these ritualscan contribute to better emotional balance and mental well being These findings open the door to more integrated approaches, recognizing religious and spiritual practices as potentially protective factors for mental health.

**Disclosure of Interest:**

None Declared

